# A Kinder Side of Red Tides?

**DOI:** 10.1289/ehp.119-a336

**Published:** 2011-08-01

**Authors:** David C. Holzman

**Affiliations:** David C. Holzman writes on science, medicine, energy, economics, and cars from Lexington and Wellfleet, MA. His work has appeared in *Smithsonian*, *The Atlantic Monthly*, and the *Journal of the National Cancer Institute*.

Investigators conducting a decade-long study into how Florida’s “red tide” algal blooms affect human health have discovered several compounds that counteract the toxins produced by the red tide dinoflagellate, *Karenia brevis*. In an intriguing twist, they are now developing one of these compounds, brevenal, as a potential treatment for cystic fibrosis (CF) and chronic obstructive pulmonary disease (COPD). In cellular and animal models brevenal appears far more potent than existing drugs for CF and COPD and exhibits the unique quality of both suppressing inflammation and boosting mucociliary clearance.^1^

The investigators spent five years identifying compounds produced by *K. brevis* that cause classic red tide symptoms of bronchoconstriction, watering eyes, and slowed mucociliary clearance. “Then we expanded the study to characterize all the degradation products, the side products, and coproducts from the biosynthetic machinery that could also be airborne, as we thought these might have some effect on people,” says Dan Baden, director of the Center for Marine Science at the University of North Carolina, Wilmington, and principal investigator for the National Institute of Environmental Health Sciences grant beind the work.

To the investigators’ surprise, three compounds actually mitigated the classical red tide toxicities. That seemed to explain a theretofore baffling observation: at certain times during a bloom, red tide toxicities appear to abate despite *K. brevis*’ abundance in the waters, Baden says.

The most promising of these compounds is brevenal.^2^ In cellular and sheep models, brevenal is therapeutically active at nanogram to picogram concentrations. In comparison, amiloride and glucocorticosteroids, used to treat CF and COPD, are active at microgram to milligram doses. Moreover, even the highest doses of brevenal tested caused no sign of irritation in the airways or other toxicity, says investigator William M. Abraham, director of research at Mount Sinai Medical Center, Miami Beach.^1^^,^^3^

Animal tests showed the compound had two effects that Abraham says are especially useful in diseases where there is impaired mucus clearance. First, it stimulated normal mucociliary clearance. Second, it blocked the mucociliary-slowing effect of human neutrophil elastase administered to the animals. Neutrophil elastase, an immune mediator, is often overproduced in respiratory diseases such as COPD and CF, making mucus thick and sticky. “The newest [unpublished] information we have is that brevenal reduces the recruitment of neutrophils to the airway in response to elastase, making brevenal a molecule that has both mucociliary and antiinflammatory activity,” Abraham says.

**Figure:**
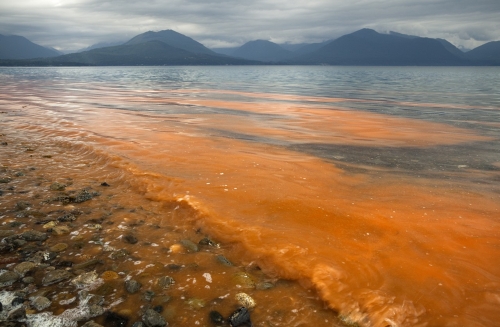
Red tide off the U.S. West Coast. © Don Paulson/SuperStock/Corbis

Baden says brevenal interacts with a previously undescribed therapeutic target.^4^ It is also the only known example of an algal compound that counteracts toxins produced by the same microorganism, according to Michael Twiner, an assistant professor of biology in the University of Michigan Department of Natural Sciences, who was not involved in the research.

Brevenal’s novelty makes it attractive from a business point of view, Baden says, and its chemistry, uses, and derivatives have been patented. Marine Biotechnology in North Carolina (MARBIONC), a unit within the Center for Marine Science, has teamed up with Ocean Therapeutics of Cary, North Carolina, to develop and commercialize the compound. Ocean Therapeutics has an exclusive license to access the library of compounds developed by MARBIONC, which Brian Dickson, Ocean Therapeutics’ chief medical officer, describes as unique in including organisms from hydrothermal vents and other locations where organisms must adapt to physiologically stressful conditions.

John E. Baatz, a professor in the Department of Pediatrics, Medical University of South Carolina, notes the animal studies used very small numbers (4–8 in most cases), and he is not sanguine about using brevenal for COPD due to multiple complex—and incompletely understood—mechanisms acting in that disease. Animal studies to date have focused on toxicologic and morphologic responses to a single dose of brevenal, he says, whereas multiple doses would likely be required to treat CF or COPD.

Furthermore, Baatz says, “[Although] cystic fibrosis is a disease for which brevenal treatment appears to be a potential treatment option, it will likely be more expensive, require multiple dosing, and not necessarily [be] more effective than other pharmaceuticals. I would need a little more persuasive data to see otherwise.”

Dickson sees a different challenge in the offing. For ethical reasons, CF drugs have to be tested in adult patients, but mucous membranes thicken with age. “Getting a drug across a thick mucosal membrane is quite difficult,” he explains. Yet, a drug that fails in an adult might well prove powerfully effective for a child because “young children [with CF] have pretty normal mucosa.” Dickson also points out that, if effective, this drug would provide only symptomatic relief for CF patients. “While this is clinically important,” he says, “most other research in this area is looking for the Holy Grail such as a treatment for the root cause.”

Generally, Dickson says, about 30% of drugs that show this much promise at this stage get to market. But if the drug, now about eight months from the end of preclinical testing, should pass muster with the U.S. Food and Drug Administration, its status as an orphan drug^5^ could result in its getting on the market within five years.
